# Combined use of 3D-printed customized prostheses and denosumab for the treatment of giant cell tumor of the distal radius: clinical outcomes and efficacy

**DOI:** 10.3389/fonc.2025.1562205

**Published:** 2025-08-18

**Authors:** Ye Li, Longqing Li, Xuanhong He, Taojun Gong, Zhuangzhuang Li, Xiaoyan Liu, Yi Luo, Yong Zhou, Li Min, Chongqi Tu

**Affiliations:** ^1^ Department of Orthopedics, Orthopaedic Research Institute, West China Hospital, Sichuan University, Chengdu, China; ^2^ Bone and Joint 3D-Printing and Biomechanical Laboratory, Department of Orthopedics, West China Hospital, Sichuan University, Chengdu, China

**Keywords:** denosumab, giant cell tumor, distal radius, 3D printing, prosthesis

## Abstract

**Objective:**

To investigate the short- and mid-term clinical efficacy of denosumab combined with 3D-printed prosthesis in the treatment of patients with giant cell tumor of the distal radius.

**Methods:**

From January 2016 to January 2022, 20 patients with giant cell tumor of the distal radius underwent denosumab treatment combined with 3D-printed prosthetic reconstruction at our hospital. This study evaluates the short- and mid-term efficacy by analyzing clinical cases where denosumab was used preoperatively, followed by 3D-printed biological prosthesis reconstruction of the distal radius tumor segment defect. We analyzed complications, function, survival rate, and recurrence rate after denosumab treatment and surgery.

**Results:**

A total of 20 patients underwent 3D-printed biological prosthesis replacement of the distal radius, with an average of 5.5 doses (range, 4-7) of 120 mg denosumab administered preoperatively. The average age of the patients was 37.2 years (range, 17–52 years), with an average follow-up of 47.3 months (range, 24–72 months). At the last follow-up, no local recurrence or pulmonary metastasis was observed in any of the patients. The pre-treatment wrist range of motion (ROM) was: extension 17.0° (range, 5°-25°), flexion 17.3° (range, 10°-30°), pronation 19.3° (range, 10°-30°), and supination 18.8° (range, 10°-30°). After denosumab treatment and before prosthesis replacement, wrist ROM improved to: extension 33.0° (range, 15°-70°), flexion 39.0° (range, 15°-60°), pronation 37.5° (range, 20°-55°), and supination 40.5° (range, 20°-60°). After prosthesis replacement, wrist ROM further improved to: extension 46.4° (range, 20°-80°), flexion 55.8° (range, 20°-85°), pronation 57.0° (range, 30°-80°), and supination 61.8° (range, 25°-80°). The average Mayo wrist score was 71.8 points, and the average Disabilities of the Arm, Shoulder and Hand (DASH) score was 16.2 points. Regarding complications, one patient experienced wrist subluxation postoperatively, and two patients experienced distal radioulnar joint separation.

**Conclusion:**

Denosumab treatment during the prosthesis production period improved wrist function and inhibited tumor progression. Patients undergoing 3D-printed biological prosthesis replacement of the distal radius showed good short- and mid-term functional outcomes, with good integration of the prosthesis with the host bone and low prosthesis-related complications. The overall clinical outcomes were satisfactory, though long-term effects of the prosthesis require further observation.

## 1 Introduction

Giant cell tumor of bone (GCTB) is a rare primary intermediate bone tumor with a local aggressive behavior (1). It typically occurs between the ages of 20 and 45, accounting for approximately 4-5% of all primary bone tumors (2). The typical site of occurrence for GCTB is the metaphysis of long bones, often extending into the epiphysis (3). While GCTB has a low metastatic rate, there is a higher risk of local recurrence post-curettage, and in rare cases, it can transform into a malignant form (4). In 1987, Campanacci et al. established a radiographically-based grading system for giant cell tumors of bone (GCT), which classifies lesions into three stages—Grade I (quiescent/benign), Grade II (active), and Grade III (aggressive)—according to key radiographic features including the degree of osteolytic destruction, margin definition, and cortical integrity (5). This system has since become a cornerstone in clinical decision-making. Treatment strategies are stage-dependent: curottage (with joint preservation) is recommended for Campanacci Grades 1–2 to achieve optimal functional outcomes, whereas Grade 3 lesions typically require en bloc resection and reconstruction (6).

The incidence rate of giant cell tumor of the distal radius GCTB accounts for approximately 10% of all cases, ranking third after the distal femur and proximal tibia (7). While it was previously believed that GCTB in the distal radius had higher invasiveness and recurrence rates, recent studies have largely refuted this notion (8). However, there is still controversy regarding surgical approaches and postoperative recurrence rates. Nevertheless, the viewpoint advocating for tumor segment resection and reconstruction is widely accepted for Campanacci III-grade or recurrent GCTB of the distal radius (9, 10). Due to the high functional demands of the wrist joint and the complex anatomy of the distal radius, reconstruction following tumor segment resection poses significant challenges (11). In recent years, various reconstruction methods have been described, such as allograft joint transplantation and total joint arthroplasty, but all have their limitations (12–14). Moreover, with the advancement of 3D printing technology, patients previously deemed ineligible for limb salvage can now undergo limb-preserving surgery through customized personalized prostheses (15). 3D-printed custom prostheses, particularly those with silver coating, have demonstrated favorable postoperative functional outcomes in bone tumors across various anatomical sites (16). In our previous research, we reported a method of wrist joint reconstruction using non-bone cement 3D-printed custom implants, which achieved better wrist function than allograft joint transplantation (17). However, it is worth noting that the production period of 3D-printed custom implants is relatively long, which may delay the treatment process and lead to the progression of GCTB.

Denosumab is a monoclonal antibody against RANKL, capable of inhibiting bone resorption, proliferation, and osteoclast activity (18). In 2013, the FDA approved Denosumab for the treatment of locally advanced or metastatic GCTB, marking the era of multidisciplinary treatment for GCTB (19). Studies have shown that preoperative use of Denosumab can achieve satisfactory local control and function, and may contribute to reducing the surgical grade. (20) However, there are also reports suggesting that preoperative adjuvant use of Denosumab may increase the risk of local recurrence of GCTB (21). Therefore, there is some controversy regarding the preoperative use of Denosumab.

Considering that the production cycle of 3D-printed customized prostheses may delay treatment, and given that some studies have reported clinical benefits of adjuvant Denosumab, our institution combines 3D-printed customized prosthesis reconstruction with adjuvant Denosumab therapy for the treatment of challenging subtypes such as distal radius GCTB, aiming to achieve better oncological outcomes and postoperative function. This study reports the short- and mid-term clinical results of this patient series.

## 2 Patients and methods

In this study, we retrospectively collected and analyzed clinical data of patients with GCTB who underwent 3D-printed uncemented endoprosthesis reconstruction of the distal radius and received adjuvant Denosumab therapy at the Musculoskeletal Tumor Center of West China Hospital from September 2015 to June 2021. The inclusion criteria were as follows: (1) age between 20 and 75 years old; (2) pathologically confirmed GCTB with Campanacci grade III or recurrent GCTB; (3) follow-up duration exceeding 24 months. Exclusion criteria were: (1) GCTB classified as Campanacci grade I-II or malignant GCTB; (2) patients who did not undergo 3D-printed uncemented endoprosthesis reconstruction; (3) lack of preoperative Denosumab therapy; (4) follow-up duration less than 24 months or incomplete follow-up data.

All patients underwent preoperative biopsy for definitive pathological diagnosis, and the surgical boundaries were determined by X-ray, 3D computed tomography (3D-CT), and magnetic resonance imaging to assess bone destruction and soft tissue involvement in the affected limb for surgical planning. Whole-body bone scan and chest CT were used to rule out distant metastasis. Simultaneously, 3D-CT scanning of the healthy side distal radius was performed to obtain anatomical data for prosthetic design. The range of motion (ROM) of the wrist for each patient was measured using a goniometer before prosthetic design, preoperatively, and postoperatively. Additionally, DASH scores and Mayo wrist scores were recorded to evaluate wrist joint function.

All prostheses are customized by our team based on the anatomical data of each patient, and meticulously modified and optimized under the guidance of Professor Tu Chongqi, the lead surgeon, using Mimics V20.0 and Geomagic Studio 2014. In terms of manufacturing, we collaborate with Beijing Chunli Zhengda Medical Equipment Co., Ltd., utilizing electron beam melting technology (ARCAM Q10plus) to create the prostheses. The entire production process takes 2 to 4 weeks, during which patients receive treatment with Denosumab to prevent disease progression. As for the materials of the prostheses, the joint surfaces are made of ultra-high molecular weight polyethylene, while the bodies are composed of titanium alloy, with hydroxyapatite coating on the pores, shafts, and stems for repairing soft tissue reconstruction, ensuring the quality and suitability of the prostheses.

All statistical analyses were performed using the R software (version 4.1.0). A two-tailed p < 0.05 was considered statistically significant.

## 3 Results

### 3.1 Patient characteristics

**Figure 1:**
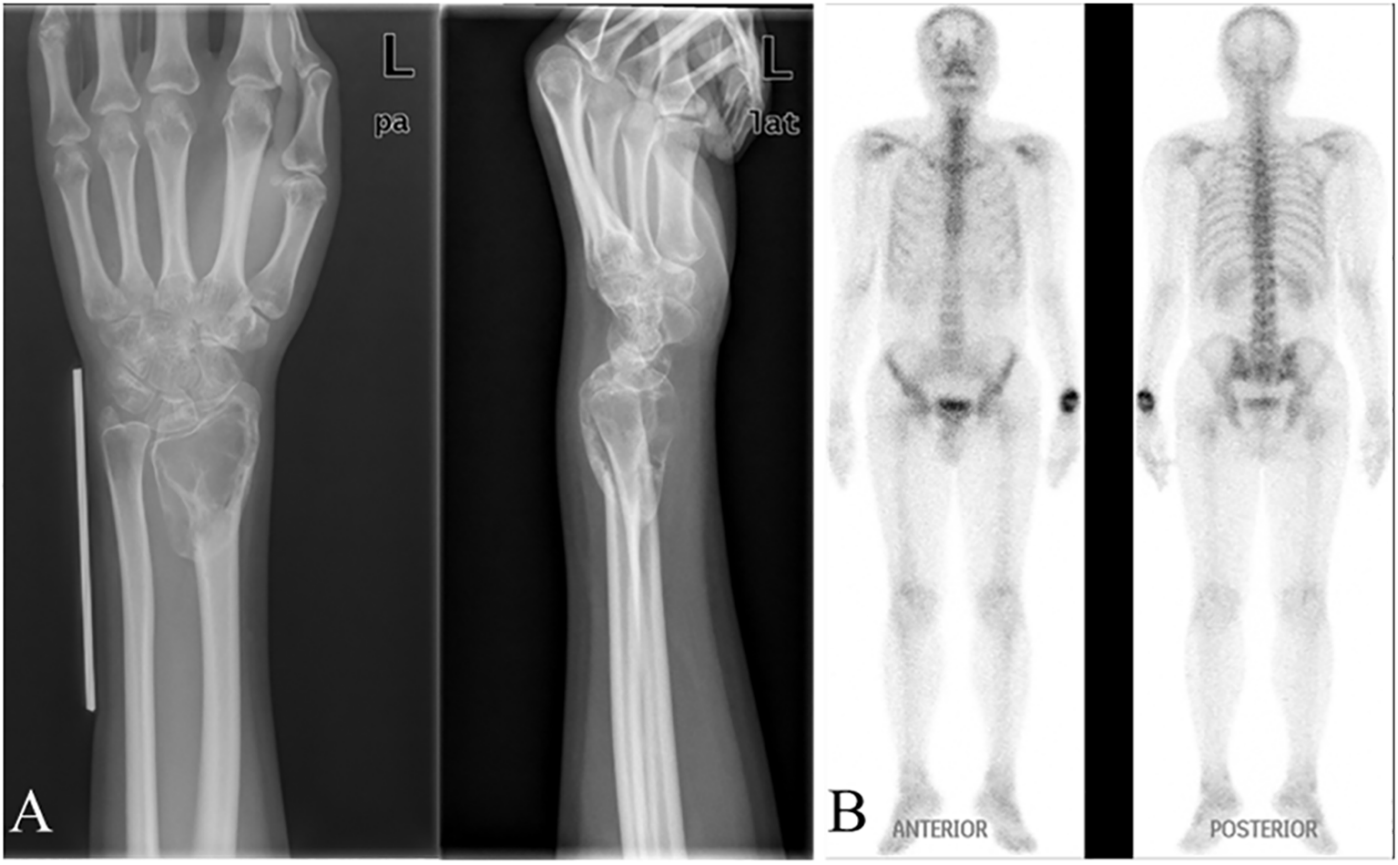
Preoperative examination results of a typical patient. A 20-year-old female patient diagnosed with a giant cell tumor of the distal left radius (Campanacci grade III), with preoperative **(A)** X-ray and **(B)** SPECT examination results.

From January 2016 to January 2022, a total of 11 patients with Campanacci III GCTB and 9 patients with recurrent GCTB met the inclusion and exclusion criteria and were included in this study. All patients underwent 3D-printed biological prosthesis replacement of the distal radius and received an average of 5.5 doses (range, 4–7 doses) of 120 mg denosumab preoperatively. Among the 20 patients, there were 10 males and 10 females, with an average age of 37.2 years (range, 17–52 years), and an average follow-up period of 47.3 months (range, 24–72 months) (**Table 1**). **Figure 1** shows the preoperative X-ray (A) and SPECT (B) of a typical patient.

**Table 1:** Preoperative demographic characteristics of 20 patients.

Patient	Age	Gender	Follow-up time	Side	Campanacci stage	Osteotomy length (cm)
1	31	Female	62	Left	III	4.5
2	43	Female	63	Right	Recurrent	5.5
3	50	Male	62	Left	III	5.0
4	35	Female	59	Left	III	4.8
5	20	Female	45	Left	III	5.4
6	41	Male	40	Left	Recurrent	6.0
7	31	Female	72	Right	III	5.5
8	43	Male	69	Left	III	4.0
9	34	Male	62	Right	Recurrent	4.5
10	43	Female	61	Left	III	4.8
11	44	Male	61	Right	Recurrent	5.0
12	41	Male	41	Left	Recurrent	6.5
13	35	Female	59	Left	III	5.0
14	44	Female	25	Right	Recurrent	7.0
15	24	Male	29	Right	Recurrent	5.5
16	25	Male	25	Right	III	5.0
17	42	Male	24	Right	Recurrent	5.8
18	62	Female	27	Left	Recurrent	7.0
19	23	Male	28	Right	III	6.5
20	34	Female	31	Right	III	6.0

### 3.2 Functional outcomes

**Figure 2:**
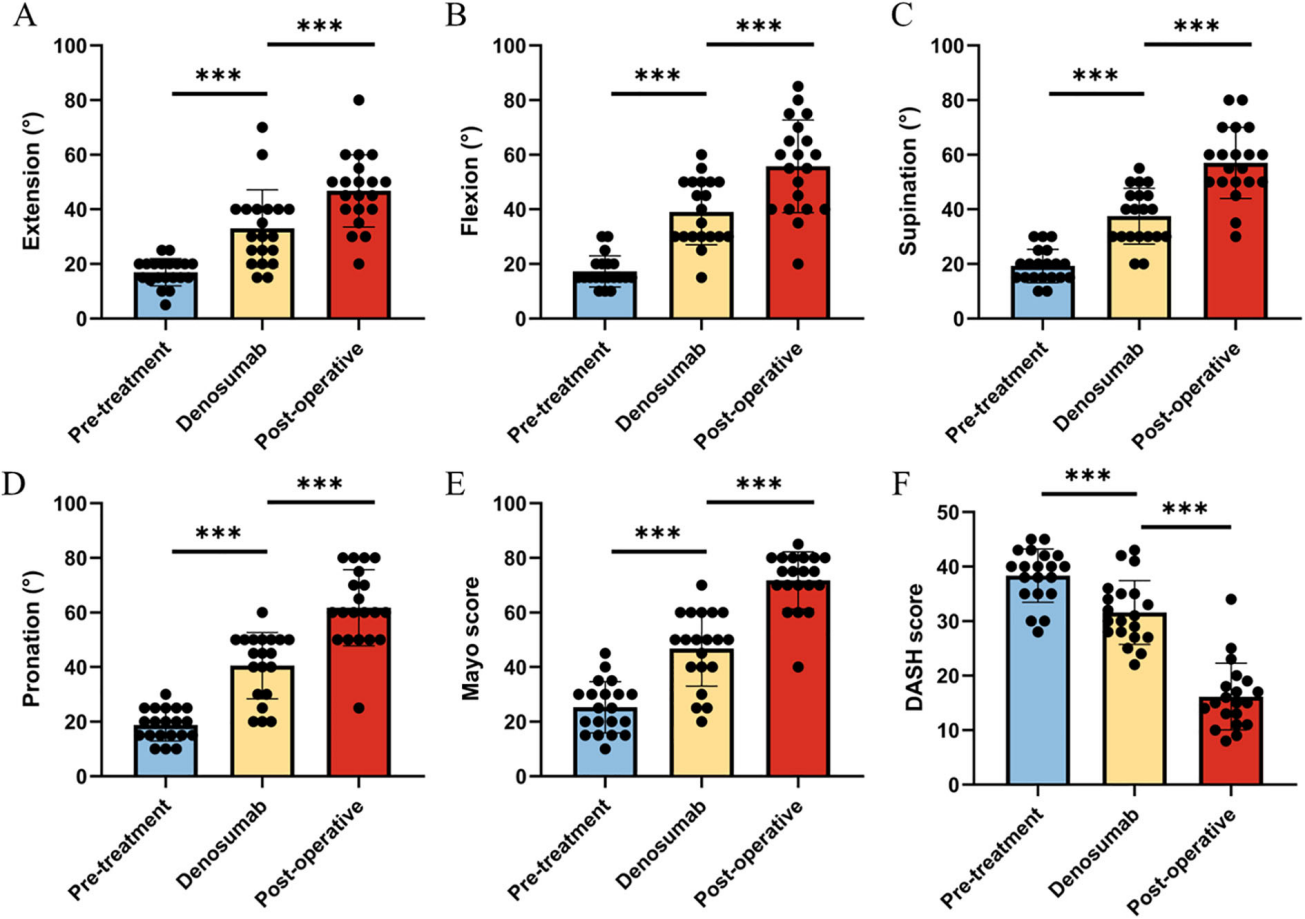
Changes in wrist joint function before treatment, after Denosumab treatment, and after surgery in the patient. **(A-D)** The range of motion in extension, flexion, supination, and pronation at three time points in the patient; **(E)** The Mayo scores of the patient at three time points; **(F)** The DASH scores of the patient at three time points.

**Figure 3:**
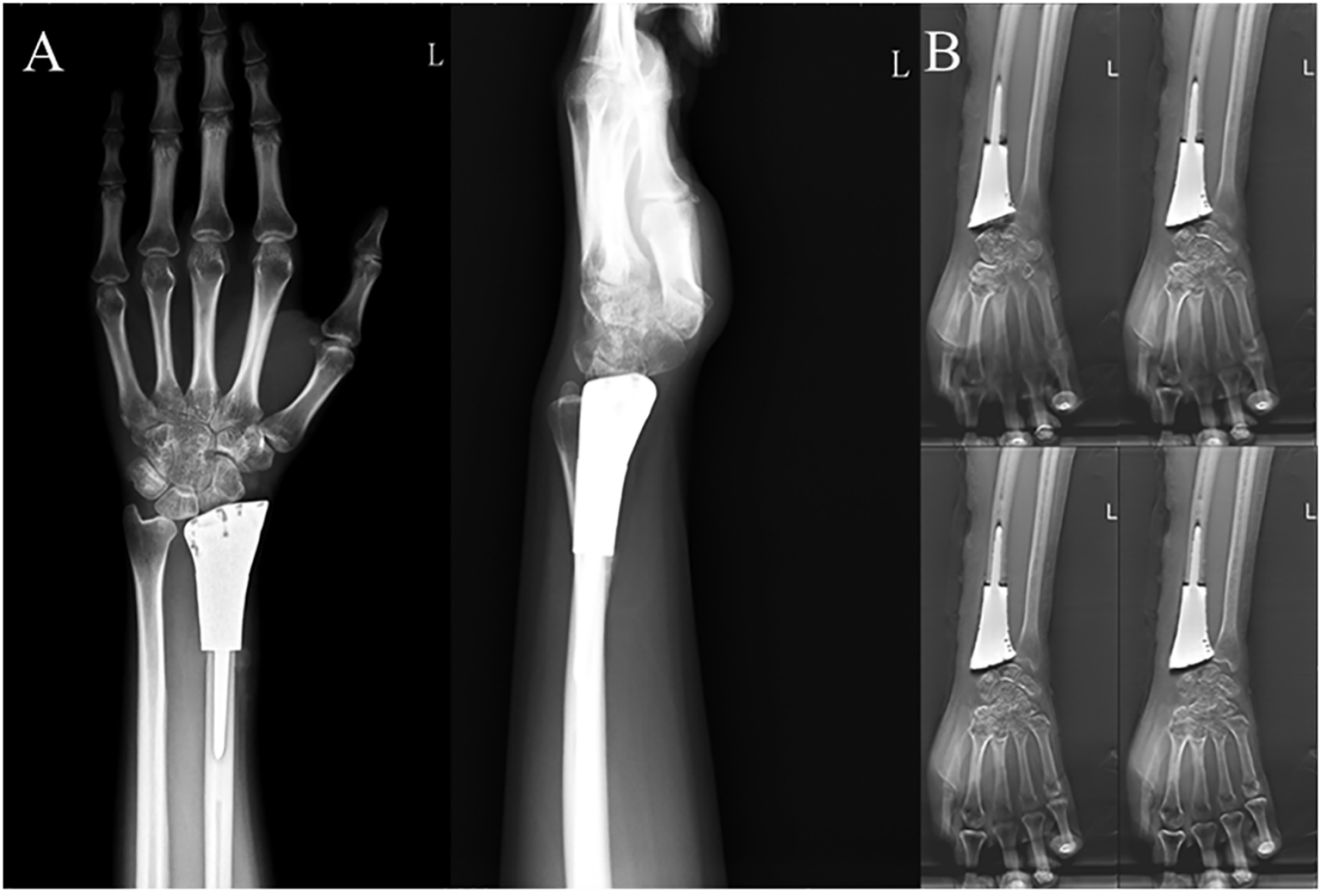
Postoperative examination results of a typical patient. **(A)** Anteroposterior and lateral X-rays of the wrist joint and **(B)** T-SMART six months after 3D-printed customized biological prosthesis replacement surgery.

**Figure 4:**
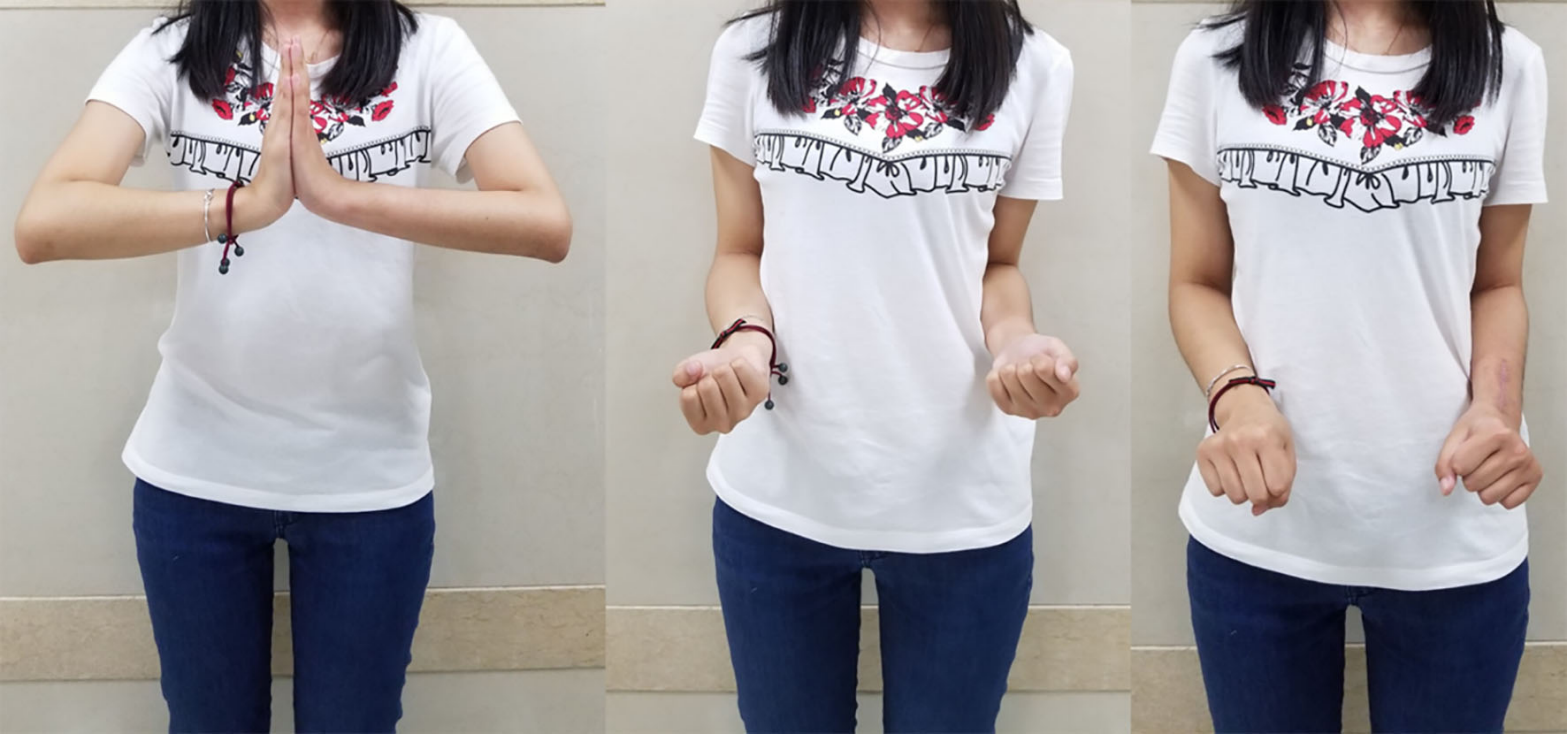
Postoperative wrist joint function images of a typical patient at 6 months. Wrist joint function images at 6 months after 3D-printed customized biological prosthesis replacement surgery.

After denosumab treatment, patients showed significant improvement in wrist ROM, which further increased following surgical treatment. Pre-treatment wrist ROM was 17.0° in extension (range, 5°-25°), 17.3° in flexion (range, 10°-30°), 19.3° in pronation (range, 10°-30°), and 18.8° in supination (range, 10°-30°). After denosumab treatment, wrist ROM improved to 33.0° in extension (range, 15°-70°, P < 0.001), 39.0° in flexion (range, 15°-60°, P < 0.001), 37.5° in pronation (range, 20°-55°, P < 0.001), and 40.5° in supination (range, 20°-60°, P < 0.001). Following surgical treatment, wrist ROM further improved to 46.8° in extension (range, 20°-80°, P < 0.001), 55.8° in flexion (range, 20°-85°, P < 0.001), 57.0° in pronation (range, 30°-80°, P < 0.001), and 61.8° in supination (range, 25°-80°, P < 0.001) (**Figures 2A–D**). Similar to wrist ROM, the pre-treatment Mayo wrist score of patients was 25.3 points (range, 10-45). After denosumab treatment, the score improved to 46.8 points (range, 20-70, P < 0.001), and further increased to 71.8 points (range, 40-85, P < 0.001) following surgical treatment (**Figure 2E**). Conversely, the DASH score of patients decreased after denosumab treatment and surgery. The pre-treatment DASH score was 38.4 points (range, 28-45). After denosumab treatment, the score improved to 31.6 points (range, 22-43, P < 0.001), and further improved to 16.2 points (range, 8-34, P < 0.001) following surgical treatment (**Figure 2F**) (**Table 2**). **Figure 3** shows the postoperative X-ray (A) and T-smart imaging (B) of a typical patient at 6 months.The patient had good wrist joint function 6 months after surgery (**Figure 4**).

**Table 2:** Wrist joint function of 20 patients before treatment, after Denosumab treatment, and after surgery.

Patient	ROM extension^*^	ROM flexion^*^	ROM pronation^*^	ROM supination^*^	Mayo wrist score^*^	DASH score^*^
1	20/40/40	10/25/45	30/40/80	15/25/75	2040/60	40/35/17
2	15/25/35	25/45/55	15/30/50	20/50/80	30/50/80	40/25/8
3	15/40/60	15/30/40	20/30/45	25/45/60	35/40/70	28/22/18
4	15/30/45	10/30/60	15/30/50	15/20/50	20/30/60	43/41/34
5	25/70/80	20/50/65	20/30/50	15/20/50	30/60/70	42/28/25
6	20/40/60	15/50/80	25/30/50	10/30/50	30/40/70	43/30/19
7	25/40/50	20/50/75	20/45/55	15/50/60	40/50/80	40/28/10
8	20/60/60	30/60/75	20/50/70	20/50/65	45/60/80	35/27/14
9	10/15/45	15/35/50	15/20/60	25/40/60	30/60/80	40/34/23
10	15/30/30	30/55/85	20/45/55	10/45/80	35/50/80	38/32/16
11	14/20/20	10/15/20	10/20/30	10/20/25	25/50/70	45/42/15
12	20/40/50	15/50/70	15/30/35	20/45/60	20/25/60	40/35/11
13	5/15/40	15/40/55	15/30/60	20/40/60	15/20/75	45/43/20
14	20/40/50	20/50/65	15/40/60	25/50/70	10/50/70	42/24/15
15	10/25/30	15/30/35	10/40/50	20/50/50	15/25/40	35/30/11
16	15/20/40	15/30/60	15/45/60	25/40/70	30/60/85	30/29/13
17	20/25/50	15/30/40	30/50/80	25/50/60	15/45/75	38/36/15
18	20/30/50	20/45/60	30/50/70	30/60/80	25/50/75	30/27/9
19	15/20/45	15/30/40	25/55/70	15/30/50	20/70/80	38/33/17
20	20/35/55	15/30/40	20/40/60	15/50/80	15/60/75	35/31/13

^*^Pre-treatment/Denosumab/Post-operative.

### 3.3 Complications

At the last follow-up, no local recurrence or pulmonary metastasis was observed in any of the patients. Among the 20 patients, one patient experienced wrist subluxation (type 1A) within one month postoperatively. Two patients had distal radioulnar joint separation; one occurred within one month postoperatively, and the other occurred within six months postoperatively. None of the included patients experienced structural failure, soft tissue failure, aseptic loosening, infection, pain, or degenerative changes as a result of the surgery.

## 4 Discussion

This study reports the short- and mid-term clinical outcomes of a multidisciplinary treatment approach for distal radius GCTB at our center. In terms of oncological outcomes, no local recurrence or distant metastasis was observed in any of the patients. Regarding functional prognosis, denosumab treatment improved wrist function, while surgical treatment further enhanced wrist range of motion.

Although previous studies have shown that reconstruction using 3D-printed custom prostheses can achieve excellent wrist function, the issue of disease control during the production period of 3D printing needs urgent resolution. Therefore, we used denosumab as a preoperative adjuvant treatment. Our results indicate that denosumab effectively controlled disease progression during the prosthesis production period. This is consistent with previous findings, as numerous clinical trials since 2010 have demonstrated favorable outcomes for denosumab in the treatment of GCTB (19, 22, 23). For instance, in the largest clinical trial to date using denosumab for GCTB, long-term follow-up revealed that most patients showed radiological response to denosumab and experienced pain relief (22).

Although some cohorts have reported that denosumab treatment may increase the recurrence rate of GCTB, these studies mostly involve patients undergoing local curettage (20, 24, 25). For example, in a retrospective analysis, the recurrence rate was 44% in patients treated with denosumab compared to 21% in the control group who did not receive denosumab. (20) The study suggested that this might be due to the newly ossified tumor matrix after denosumab treatment, which could confuse the true surgical margins (26). However, in our study, all patients underwent en bloc resection and custom prosthesis reconstruction. In the complex anatomical structure of the wrist, the sclerotic tumor margins and bone shell post-denosumab treatment facilitated the complete resection of the tumor and did not increase the recurrence rate. This also provides new evidence supporting the preoperative neoadjuvant use of denosumab in patients undergoing en bloc resection for GCTB.

In our study, we found that neoadjuvant denosumab treatment led to significant improvement in wrist function, which is consistent with previous studies. For example, in a Phase II clinical trial, 30 out of 35 patients showed functional improvement after 25 weeks of denosumab treatment (23). Similarly, in a retrospective study involving 18 patients, all patients exhibited pain relief, improved mobility, and enhanced function (21). This could be attributed to the tumor reduction and alleviation of pain symptoms following denosumab treatment.

Consistent with our previous reports, 3D-printed custom prosthesis reconstruction resulted in good wrist function for patients. However, due to baseline functional differences between patients who received neoadjuvant denosumab treatment combined with custom prosthesis reconstruction and those who did not receive denosumab treatment, the two groups are not directly comparable (17). Nonetheless, postoperative wrist function was similar between the two groups. Undoubtedly, Denosumab treatment alleviated the patient’s symptoms and inhibited tumor progression during the waiting period. This is also in line with previous findings. For instance, in a Phase II clinical trial involving 222 patients, after a median treatment duration of 19.5 months, 96% of originally planned joint replacement surgeries and 86% of planned joint fusion surgeries were converted to joint-preserving surgeries.

It should be noted that our study has certain limitations. First, our study is retrospective and may have selection bias, and the number of patients included is relatively small, which may be due to the restriction of including only patients who underwent custom prosthesis reconstruction for wrist function. Second, due to baseline functional differences between patients included in this study and those at our center who underwent custom prosthesis reconstruction without denosumab treatment, postoperative function was not compared between the two groups. Third, the follow-up period for patients is relatively short, and the long-term safety and efficacy of adjuvant denosumab treatment require further investigation. Future research necessitates a focus on long-term surveillance of implant durability and the extended safety and efficacy of adjuvant denosumab therapy.

## 5 Conclusion

This is the first study to report objective functional outcomes and complications in patients with Campanacci grade III and recurrent distal radius GCTB treated with neoadjuvant denosumab combined with custom prosthesis reconstruction. Our results indicate that denosumab treatment during the prosthesis production period improved wrist function and inhibited tumor progression. Patients who underwent 3D-printed biological prosthesis replacement of the distal radius showed good short- to mid-term postoperative function, with good healing at the prosthesis-host bone interface and low prosthesis-related complications, resulting in overall satisfactory clinical outcomes. The long-term effects of the prosthesis require further observation.

## Data Availability

The raw data supporting the conclusions of this article will be made available by the authors, without undue reservation.
